# Mapping the Outbreaks of Dengue and Chikungunya and Their Syndemic in India: A Comprehensive Analysis Over the Past Decade Utilizing the Data From the Integrated Disease Surveillance Programme (IDSP)

**DOI:** 10.7759/cureus.77193

**Published:** 2025-01-09

**Authors:** Asokan Dinesh, Siva Prasad Reddy Bommu, Aishwarya Balakrishnan, Minal Borode, Joyce Bardeskar, Ajith Ramalingam, Anjali Mall, Geeta Pardeshi

**Affiliations:** 1 Department of Community Medicine, Grant Government Medical College and Sir Jamshedjee Jeejeebhoy (JJ) Group of Hospitals, Mumbai, IND

**Keywords:** chikungunya, dengue, gis, syndemic, vector-borne diseases

## Abstract

Background: This study examines the geographic distribution, temporal trends, and syndemic interactions between dengue and chikungunya outbreaks in India from 2014 to 2023, using data from the Integrated Disease Surveillance Programme (IDSP). Understanding trends offers critical insights to enhance public health responses to co-occurring vector-borne diseases.

Objectives: The study analyzes the trends, geographic distribution, and syndemic interactions between dengue and chikungunya. It highlights patterns, high-burden areas, and seasonal dynamics to inform public health interventions for mitigating the dual burden.

Methods: This secondary data analysis used IDSP records from January 2014 to December 2023. Temporal trends in outbreaks were analyzed, and districts were categorized by severity through spatial mapping and bivariate analysis, employing statistical quartiles to assess the co-occurrence and syndemic nature of dengue and chikungunya.

Results: Dengue outbreaks increased significantly from 2014 to 2017, peaking at 175 outbreaks, followed by fluctuations until a peak of 200 outbreaks in 2023. In contrast, chikungunya outbreaks peaked in 2017 at 77 outbreaks and subsequently declined to 19 outbreaks by 2023. Geographic analysis indicated a high dengue burden in districts such as East Siang (Arunachal Pradesh), Birbhum (West Bengal), and Pune (Maharashtra), while chikungunya was most severe in Thanjavur, Theni, and Vellore (Tamil Nadu). A bivariate choropleth analysis identified regions with significant syndemic, such as Pune (Maharashtra), Tumkur (Karnataka), and Kamrup (Assam), which were classified as high-high. Additional districts such as Sangli and Satara (Maharashtra) were categorized as high-moderate, while Puducherry and Junagadh (Gujarat) were noted as moderate-high for dengue and chikungunya, respectively.

Conclusions: The findings underscore significant variability in dengue and chikungunya syndemic across India, highlighting regions with severe dual burden. Enhanced surveillance for close watch on syndemic occurrence and its impact needs to be studied, which is essential to reduce both the incidence and study its compounded public health impacts.

## Introduction

A syndemic occurs when two or more concurrent or sequential epidemics interact within a population, amplifying the disease burden through shared social, environmental, or biological pathways. These interactions are not merely additive but synergistic, compounding adverse health outcomes. Prominent examples include the syndemic of HIV and tuberculosis or malnutrition and infectious diseases in resource-constrained settings. This framework highlights the necessity of integrated strategies for managing interlinked health crises.

Dengue and chikungunya, both transmitted by the *Aedes aegypti* mosquito, exemplify a syndemic in the Indian context due to their overlapping transmission dynamics and clinical presentations. Dengue, known for its potential to cause hemorrhagic fever and severe morbidity, and chikungunya, associated with debilitating arthralgia, are frequently coendemic [1]. Coinfections exacerbate clinical outcomes, complicating diagnosis and management. Urbanization, inadequate vector control, and climate change have further intensified the spread of these arboviral diseases [2].

Geographic information systems (GIS) have emerged as critical public health tools for understanding the spatial dynamics of diseases. GIS enables the visualization of disease patterns, the identification of hotspots, and the formulation of targeted interventions. Studies have demonstrated its efficacy in mapping outbreaks of dengue and chikungunya, providing insights that inform resource allocation and policy formulation. The integration of GIS-based spatial analytics into vector-borne disease surveillance enhances predictive capabilities and outbreak preparedness [3].

This study addresses a crucial gap in understanding the spatial and temporal interactions of dengue and chikungunya outbreaks in India. Despite extensive outbreak data, limited research has explored their syndemic nature at a national scale. By leveraging GIS and a decade of Integrated Disease Surveillance Programme (IDSP) data, this study aims to identify syndemic hotspots, thereby informing strategies for mitigating these coendemic diseases compounded public health burden.

## Materials and methods

Study design and data source

This study conducted a comprehensive record-based secondary data analysis to investigate the trends, geographic distribution, and syndemic interactions of dengue and chikungunya in India from January 2014 to December 2023. We systematically analyzed data sourced from the IDSP, which compiles weekly outbreak reports. This decade-long analysis includes data spanning all Indian states and territories, ensuring a nationwide coverage of reported outbreaks.

Inclusion and exclusion criteria

The inclusion criteria for the study encompassed all confirmed weekly outbreak reports of dengue and chikungunya during the specified study period. We excluded any datasets that were incomplete or inaccessible due to technical issues within the IDSP database, ensuring the integrity and completeness of the data analyzed.

Analytical tools and techniques

Data analysis was performed using R software, version 4.3.2 (R Foundation, Vienna, Austria), which facilitated the use of advanced geospatial techniques to assess and visualize the data. The following techniques were employed.

Choropleth Maps

These maps were utilized to delineate and visualize the geographic distribution of disease outbreaks across the districts of India, highlighting areas with varying disease severity.

Bivariate Choropleth Maps

These maps were crucial for identifying geographic hotspots and assessing the syndemic interplay between dengue and chikungunya. This dual-layered approach provided insights into districts experiencing concurrent high burdens of both diseases, aiding in the identification of syndemic hotspots.

Ethical considerations

Given the secondary nature of the publicly available IDSP data, ethical approval was not required for this study. However, our methodological approach was rigorously designed to adhere to ethical principles of data usage, ensuring transparency in data handling and analysis. All data were anonymized and aggregated, eliminating any personal health information to uphold confidentiality and comply with ethical standards in public health research.

## Results

**Figure 1:**
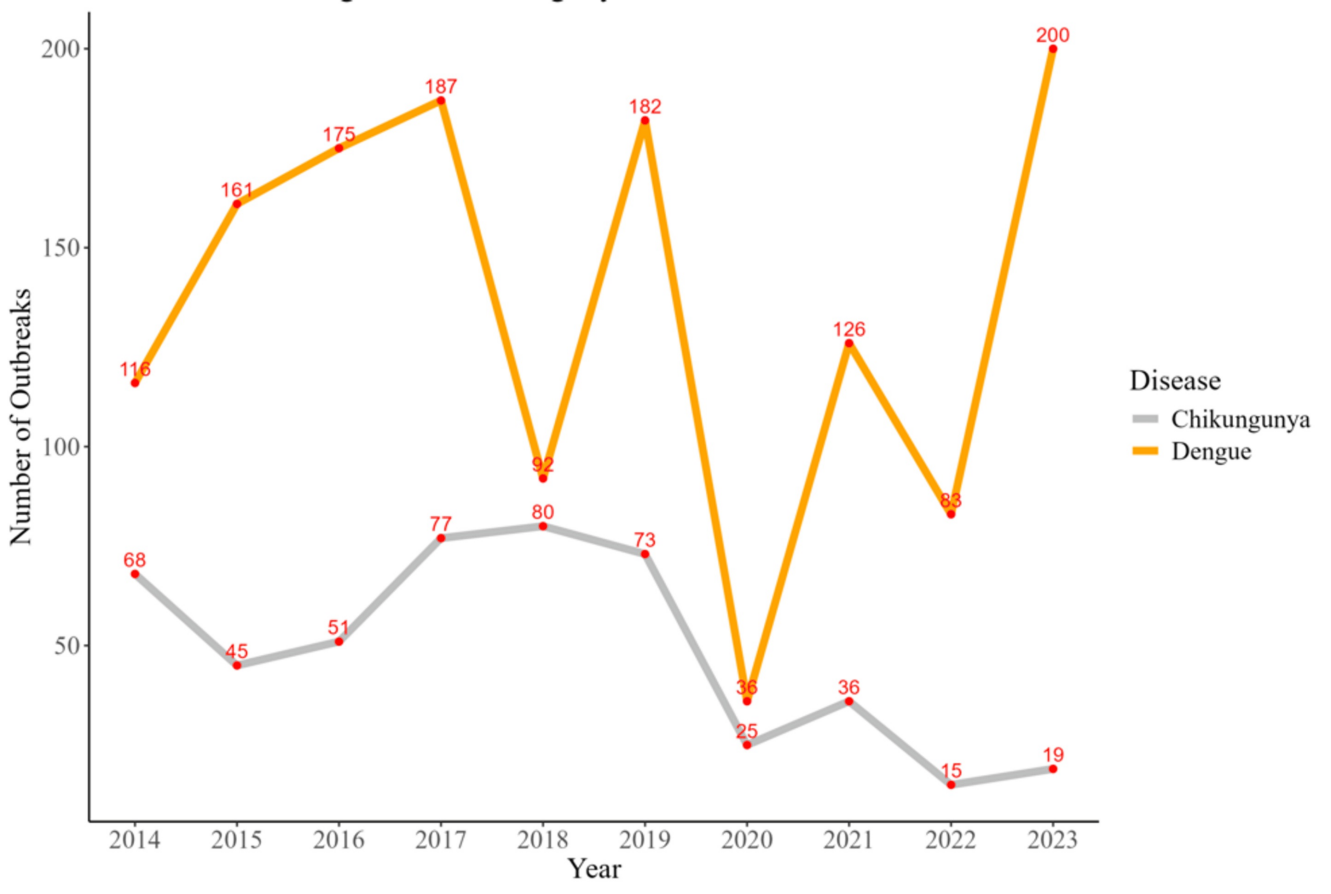
Trends in dengue and chikungunya outbreaks in India (2014-2023)

Figure 1 illustrates the temporal trends in dengue and chikungunya outbreaks across India over the study period. The line plot shows a marked increase in dengue outbreaks from 2014 to 2017, peaking at 175 outbreaks. Following fluctuations, the number of outbreaks peaked again in 2023 at 200. Chikungunya outbreaks exhibited a different trend, peaking at 77 outbreaks in 2017 and subsequently declining to just 19 in 2023. These contrasting patterns suggest a more episodic and variable nature for dengue, while chikungunya has shown a steady decline in outbreaks over the years. The cutoff values were calculated by aggregating annual outbreak counts reported by IDSP. These values were plotted to visualize temporal trends, highlighting years with significant spikes. The results emphasize the need for targeted public health interventions during peak outbreak periods.

**Figure 2:**
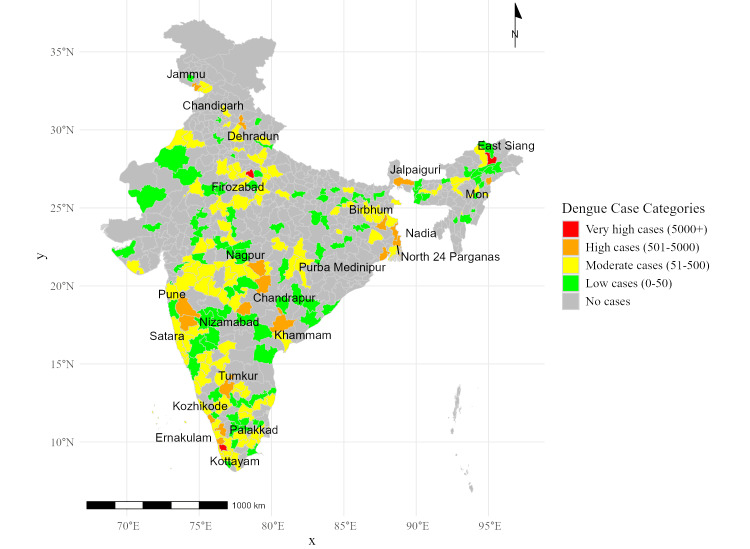
Heatmap of dengue cases by districts in India (2014-2023)

Figure 2 maps the geographic distribution of dengue cases across Indian districts, categorized by severity levels. The cutoff values were derived from statistical quartile analysis, dividing the data into four categories: very high (5,000+ cases), high (501-5,000 cases), moderate (51-500 cases), and low (0-50 cases). These thresholds were chosen to ensure that districts with similar case burdens were grouped together for targeted interpretation. The map was plotted using the R software and highlights the following findings. Districts such as East Siang (Arunachal Pradesh), Pune and Satara (Maharashtra), and Birbhum (West Bengal), which represent areas with extreme Dengue outbreaks, fell in the very high case (5,000+) category. Firozabad (Uttar Pradesh) and Dehradun (Uttarakhand), which displayed substantial dengue activity, fell in the high case (501-5,000) range. Districts like Ernakulam (Kerala) and Khammam (Telangana) fell in the moderate case (51-500) range. Minimal dengue activity was observed in districts like Tumkur (Karnataka) and Chandigarh (Union Territory), which fell in the low case (0-50) category. The map underscores regional variations, emphasizing hotspot districts for targeted interventions.

**Figure 3:**
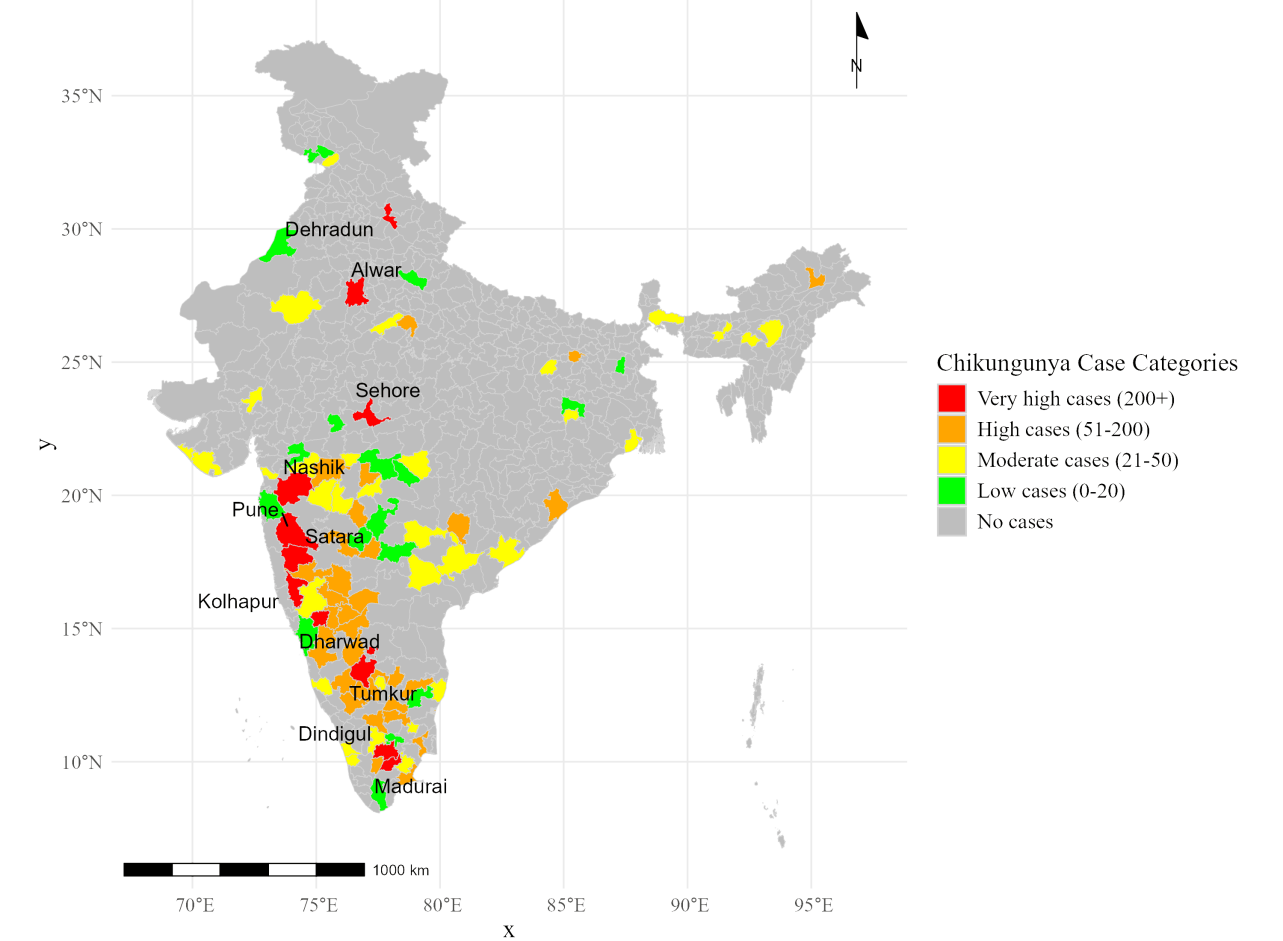
Heatmap of chikungunya cases by districts in India (2014-2023)

Figure 3 visualizes the chikungunya case distribution across districts, divided into severity categories. The cutoff values for classification were determined using interquartile ranges, with very high (200+ cases), high (51-200 cases), moderate (21-50 cases), and low (0-20 cases) categories. These thresholds were selected to distinguish areas with varying disease burdens. The map was generated using R software and revealed the following. Districts such as Thanjavur and Madurai (Tamil Nadu), and Kalaburagi (Karnataka) experienced the highest burden and fell under the very high cases (200+) category. Districts like Bijapur (Karnataka) and Parbhani (Maharashtra) reported significant outbreaks and fell under high cases (51-200). Nalanda (Bihar) and Akola (Maharashtra) reflected moderate case counts and fell under the moderate case (21-50) category. Dehradun (Uttarakhand) and Sehore (Madhya Pradesh) showed minimal chikungunya activity, which fell under the low case (0-20) category. This visual representation highlights regions requiring enhanced surveillance and vector management efforts.

**Figure 4:**
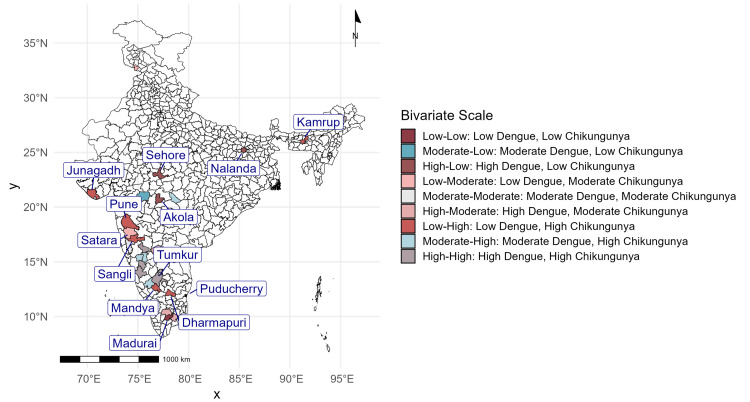
Bivariate choropleth map assessing the syndemic interplay between dengue and chikungunya (2014-2023)

Figure 4 presents a bivariate choropleth map, overlaying dengue and chikungunya data to identify dual-burden districts. The cutoff values for classification were based on quartile combinations for both diseases, resulting in nine categories, of which high-high, high-moderate, and moderate-low represent a higher burden. These classifications ensured clear differentiation of regions based on the dual burden of the diseases. The key findings include the following: Pune (Maharashtra), Tumkur (Karnataka), and Kamrup (Assam) were critical hotspots for both diseases and fell under the high-high burden category. Regions like Sangli and Satara (Maharashtra) reported high dengue cases alongside moderate chikungunya activity, which fell under the high-moderate burden category. Puducherry and Junagadh (Gujarat) displayed the opposite pattern, with high chikungunya and moderate dengue activity, which fell under the moderate-high burden category. This dual-burden analysis facilitates the prioritization of resources and integrated intervention strategies, highlighting regions where simultaneous action against both diseases is necessary.

## Discussion

The temporal trends observed in dengue and chikungunya outbreaks highlight the dynamic nature of vector-borne diseases in India over the past decade. The steady rise in dengue outbreaks from 2014 to a peak in 2023 underscores the growing challenges posed by urbanization, population density, and changing climatic conditions. These factors have been widely documented as drivers of *A. aegypti* proliferation, as seen in studies by Bhatt et al. [4]. Meanwhile, the decline in chikungunya outbreaks after 2017 may suggest a degree of acquired population immunity or the effectiveness of vector control measures in some regions, consistent with findings by Mourya et al. [5]. Similar cyclical trends in arboviral diseases have been observed globally, with outbreaks often peaking during favorable climatic conditions, as documented by Lambrechts et al. [6]. This emphasizes the need for sustained vector surveillance and preemptive public health interventions to manage outbreaks effectively.

Geographically, dengue was found to be more concentrated in urban and densely populated districts like Pune (Maharashtra), East Siang (Arunachal Pradesh), and Birbhum (West Bengal). These findings align with earlier studies that highlighted the role of urban environments, unplanned development, and poor water management in creating optimal conditions for mosquito breeding [7]. Conversely, chikungunya displayed a distinct geographic distribution, with high-burden districts concentrated in Tamil Nadu, such as Thanjavur and Theni, and parts of Karnataka. This regional variation underscores the need for tailored intervention strategies that address specific environmental and sociodemographic risk factors.

The bivariate choropleth analysis offers a clear understanding of the syndemic interaction between dengue and chikungunya, revealing critical dual-burden hotspots like Pune (Maharashtra), Tumkur (Karnataka), and Kamrup (Assam). These findings reflect the compounded public health challenges posed by coendemic arboviral diseases, which amplify morbidity through overlapping transmission cycles and clinical presentations. Studies such as that by Carlson and Mendenhall [8] have emphasized the syndemic framework, demonstrating how the interaction between multiple epidemics worsens health outcomes beyond their individual effects. Coinfections, as reported by Furuya-Kanamori et al. [9], complicate diagnosis and treatment, leading to delayed recovery and increased healthcare costs. Additionally, high-moderate and moderate-high districts such as Sangli and Junagadh highlight the geographic heterogeneity of this syndemic, necessitating region-specific strategies.

Limitations

The study's reliance on IDSP data introduces limitations such as underreporting, incomplete rural data, and variability in state-level quality, potentially affecting hotspot identification. The exclusion of environmental factors like rainfall and temperature, along with sociodemographic data, restricts insights into ecological drivers and vulnerability to syndemics. These gaps highlight the need for improved, comprehensive surveillance systems.

Recommendations

Based on the study findings, establishing syndemic surveillance systems in hotspots like Pune, Satara, Sehore, Tumkur, and Kamrup is essential for monitoring and addressing the dual burden of dengue and chikungunya. Integrating GIS-based mapping into IDSP operations can enhance hotspot identification and streamline resource allocation. Premonsoon vector control campaigns in high-burden districts should prioritize localized interventions, including eliminating mosquito breeding sites and improving water storage practices. Community awareness programs in regions like Pune and Tumkur must educate residents on prevention and coinfection risks to foster active participation in vector control efforts. Strengthening healthcare infrastructure in these areas is crucial for early diagnosis and effective management of coendemic diseases. Dual-disease control strategies, such as larvicide application and community engagement, can effectively address both diseases in high-risk regions. Additionally, predictive modeling based on outbreak trends can identify districts vulnerable to future outbreaks, enabling timely interventions. Coordinated efforts between state and district health authorities in syndemic-prone regions like Kamrup and Puducherry will ensure efficient resource utilization and mitigate the compounded impact on public health.

## Conclusions

The study highlights the evolving epidemiology of dengue and chikungunya outbreaks in India, with clear geographic and temporal variabilities. Dengue outbreaks peaked in 2016, 2018, and 2021, reflecting periodic surges, while chikungunya exhibited a declining trend after 2018. Districts such as East Siang, Firozabad, and Kottayam emerged as hotspots for dengue, while chikungunya was highly concentrated in regions like Pune, Tumkur, and Sehore. Syndemic hotspots, where both diseases co-occurred, were identified in key districts such as Pune, Satara, Kamrup, and Puducherry, underscoring the amplified public health burden in these areas. The study underscores the significant impact of overlapping disease burdens in high-burden districts, where coendemic outbreaks exacerbate morbidity and strain healthcare resources. This research provides an evidence-based framework to guide public health officials in mitigating the compounded impacts of dengue and chikungunya through integrated and region-specific strategies.
